# Draft Genome Sequence of *Lactobacillus plantarum* BFE 5092 Isolated from Maasai Fermented Milk

**DOI:** 10.1128/genomeA.00481-16

**Published:** 2016-06-02

**Authors:** Folarin A. Oguntoyinbo, Gyu-Sung Cho, Erik Brinks, Gregor Fiedler, Jan Kabisch, Sabrina Koberg, Wilhelm Bockelmann, Horst Neve, Youn-Goo Kang, Doyeon Yun, Ah-Ram Kim, Arjan Narbad, Charles M. A. P. Franz

**Affiliations:** a Department of Microbiology and Biotechnology, Max Rubner-Institut, Federal Research Institute of Nutrition and Food, Kiel, Germany; b Department of Microbiology, Faculty of Science, University of Lagos, Lagos, Nigeria; c School of Life Science, Handong Global University, Pohang, South Korea; d Gut Health and Food Safety Program, Institute of Food Research, Norwich, United Kingdom

## Abstract

The draft genome of *Lactobacillus plantarum* BFE 5092 isolated from the Maasai traditional fermented milk product kule naoto was sequenced, and sequence analysis showed the assembled genome size to be 3,285,094 bp, containing a predicted total of 3,111 protein-encoding genes, 17 rRNAs, and 70 tRNAs.

## GENOME ANNOUNCEMENT

*Lactobacillus plantarum* is a lactic acid bacterium that has been isolated from diverse ecosystems, such as vegetables, meat, fish, dairy products, and the gastrointestinal tract (1). This species is known for its high phenotypic, genomic, and metabolic diversity, the consequences of which are central to the success of its industrial application. This versatility of the species is largely based on the presence of so-called genomic “lifestyle islands” that consist of numerous functional gene cassettes, particularly for carbohydrate utilization, which can be acquired, shuffled, substituted, or deleted in response to niche requirements (1).

*L. plantarum* BFE 5092 was isolated from a fermented sour milk product, kule naoto, produced by the Maasai in Kenya. The strain is considered to have potential as a probiotic strain, as it was found to survive gastrointestinal passage in an *in vitro* model, showed antimicrobial activity toward and coaggregation with pathogens, and adhered well to HT29 MTX cells in cell culture (2 – 4). The strain was also shown to possess genes for the production of two plantaricins (i.e., EF and JK) (5) and is currently investigated for its potential as a starter culture for the fermentation of African leafy vegetables. Currently, there are genome sequences of 34 *L. plantarum* strains available, of which 7 have been completed. The genome of strain BFE 5092 was sequenced in order to assess its technological and functional properties and to compare its genome sequence with those of other sequenced *L. plantarum* strains from different sources.

The genomic DNA of *L. plantarum* BFE 5092 was isolated using the Qiagen Genomic-tip 100/G kit (Qiagen, Manchester, United Kingdom). The library was prepared with an Illumina Nextera XT library prep kit (Illumina, San Diego, CA), and genome sequencing was done with an Illumina MiSeq sequencer. In total, 2,909,131 paired-end sequence reads of 500 bp in length were obtained with 117-fold coverage. The template-based assembly was performed using Andrew And Aaron’s Awesome Assembly pipeline (A5-miseq, version 20140604), which yielded 66 scaffolds. The largest scaffold was 425,807 bp in size. The *N*_50_ was 122,578 bp. The genome of *L. plantarum* BFE 5092 is 3,285,094 bp in size, with a 44.39 mol% G+C content. The genome sequence was annotated using the Rapid Annotations using Subsystems Technology (RAST) server (http://rast.nmpdr.org/) (6, 7). The genome contains 3,111 protein-coding genes, 17 rRNAs, and 70 tRNAs. Four hundred thirty-three genes are involved in sugar metabolism, including genes for 11 phosphoenolpyruvate (PEP)/phosphotransferase systems (PTS) for the utilization of *N*-acetylglucosamine, mannose, sucrose, maltose, and glucose, beta-glucoside sugars, cellobiose, trehalose, mannitol, galactitol, fructose, as well as glucitol and sorbitol sugars. The genome information for this wild-type strain will be useful for its further development and application as a multifunctional starter culture.

### Nucleotide sequence accession number.

This whole-genome shotgun project has been deposited at the European Nucleotide Archive (ENA) under the accession no. FJVL01000000.
